# Gentle Manual Acupuncture Could Better Regulate Gastric Motility and Vagal Afferent Nerve Discharge of Rats with Gastric Hypomotility

**DOI:** 10.1155/2019/9043151

**Published:** 2019-10-24

**Authors:** Yangyang Liu, Yang Bai, Yue Pan, Zhifang Xu, Yuxin Fang, Shenjun Wang, Yuanhao Du, Yi Guo

**Affiliations:** ^1^Research Center of Experimental Acupuncture Science, Tianjin University of Traditional Chinese Medicine, No. 10, Poyang Lake Road, Tuanbo New Town, Jinghai District, Tianjin 301617, China; ^2^Acupuncture and Massage Department, Qingyang Hospital of Traditional Chinese Medicine, No. 10, Guxiang West Road, Qingyang City, Gansu 745000, China; ^3^Acupuncture Department, The First Affiliated Hospital of Tianjin University of Traditional Chinese Medicine, No. 88, Changling Road, Xiqing District, Tianjin 300391, China; ^4^Chinese Medical College, Tianjin University of Traditional Chinese Medicine, No. 10, Poyang Lake Road, Tuanbo New Town, Jinghai District, Tianjin 301617, China

## Abstract

The variation of stimulus intensity of manual acupuncture (MA) may produce diverse acupuncture effects. However, the intensity-effect relationship and the underlying mechanism of MA are still elusive. In this study, the effects of MA regulation of gastric motility were investigated after lifting-thrusting MA treatment with four different frequencies (1 Hz, 2 Hz, 3 Hz, and 4 Hz) at ST36. The experiments were conducted on rats with gastric hypomotility caused by atropine. The results showed that the gastric motility amplitude decreased after atropine injection, while the treatment of four types of MA affected the gastric motility amplitude in varying degrees. Specifically, 2 Hz MA exhibited the most effective results, while 4 Hz MA had the least effect; the effects of 1 Hz MA and 3 Hz MA were between the effects induced with 2 Hz and 4 Hz. Furthermore, the response of gastric vagal afferent nerve discharge and gastric motility was examined after MA treatment with frequencies of 2 Hz and 4 Hz, respectively, on ST36 in order to elucidate the mechanism of MA regulation of gastric motility. The results showed that 2 Hz MA was able to increase the amplitude of gastric motility and discharge frequency of gastric vagal afferent nerves, while 4 Hz MA exhibited seldom effects. These findings suggest that gentle MA (2 Hz) has more stimulating effects than strong stimulation with MA (4 Hz) on gastric hypomotility. In addition, gastric motility regulated by MA was associated with vagal afferent nerve activation.

## 1. Introduction

Acupuncture originates from ancient China and has been applied for more than 3000 years [1]. Due to its efficiency and safety, acupuncture is currently widely used in health care systems all over the world [2, 3]. Acupuncture treatments are complex multicomponent interventions, and they include needling components, specific (acupuncture theory-related) nonneedling components, and nonspecific nonneedling components (i.e., therapeutic setting, time, and attention) [4]. Regarding needling components, needling stimulation intensity is an important factor, which may influence the therapeutic effects of acupuncture. Manual acupuncture (MA) is the traditional, wildly used acupuncture technique, which implies treating patients by inserting thin, solid needles into acupuncture points (acupoint) on the skin. The needles are often manipulated by the practitioner to elicit the de-qi sensation (i.e., pain, achiness, stinging, or dullness at the needle insertion site) and to induce the therapeutic effects [5, 6]. The most commonly used manual manipulations are lifting-thrusting and twirling manipulation [7]. Variations in frequency, forcefulness, and amplitude of the manipulations may produce different stimulation types (e.g., duration and intensity), and thereby affect the impact of acupuncture treatment [8]. However, the intensity-effect relationship of manual acupuncture and its mechanism remains unclear. There is a lack of available guidelines on how to select appropriate manual manipulation parameters in acupuncture clinical practice.

Gastric hypomotility, associated with delayed gastric emptying, may result in abdominal pain, nausea, vomiting, heartburn, loss of appetite, and other digestive problems. Furthermore, gastroesophageal reflux disease, gastritis, nonulcer or functional dyspepsia, and gastroparesis are commonly encountered disorders of gastric motility in clinical practice. It has been reported that acupuncture can alleviate multiple symptoms of gastrointestinal disorders [9–15], and the “Zusanli” (ST36) is the most commonly used acupoint to treat gastric disorders [16–21]. In addition, the gastric function is mainly regulated by the parasympathetic nervous system, especially the gastric vagal nerves. Gastric vagal afferent nerves play an integral part in conveying information from the stomach to nucleus tractus solitaries (NTS) which is relevant for regulation of certain gastrointestinal functions (e.g., secretion, storage, propulsion, and emptying), while vagal efferent nerves are responsible for transmission of regulatory signals from the dorsal motor nucleus of the vagus (DMV) to the stomach [22]. Therefore, the activity of the gastric vagal nerve can affect and regulate the gastric function.

In the present study, rats with gastric hypomotility caused by atropine were used to test the effect of gastric motility regulation by lifting-thrusting MA with frequencies of 1 Hz, 2 Hz, 3 Hz, and 4 Hz on ST36, respectively. The effects of variable stimulus intensity were examined in order to elucidate the correlation between the intensity of the stimulus and the therapeutic impact of MA. After the detection of distinctive effects between different MA frequencies, the most effective and the least effective frequency were applied to study the mechanism of gastric vagal afferent nerve activity.

## 2. Materials and Methods

### 2.1. Animal Preparations

The experiments were performed on healthy adult male SD rats (250–280 g of weight), provided by the Laboratory Animal Centre of the Academy of Chinese Military Medical Sciences (animal license number: SCXK (Jin 2009-0004)). All manipulations and procedures were carried out in accordance with the Guidance Suggestions for the Care and Use of Laboratory Animals of the Ministry of Science and Technology of China and were approved by the Animal Ethics Committee of the Tianjin University of Traditional Chinese Medicine. The rats were housed in groups at 23 ± 1°C and maintained under a 12-hour light/dark cycle with food and water available ad libitum. After an overnight fast of 18 h, the rats were anesthetized with urethane (1.5 mg/kg, i.p.). A tracheal cannula was inserted to facilitate spontaneous breathing. Body temperature was maintained at 37.5°C with a feedback-controlled heating pad.

### 2.2. Gastric Motility Recording

Gastric motility recording was performed as described previously by Gao et al. [23]. The abdomen was opened by a longitudinal incision (5–6 cm in length) right below the diaphragm. The stomach was exposed entirely. A balloon (0.5 cm in diameter) attached to a catheter (1.5 mm OD and 1.1 mm ID) was inserted into the stomach by making a small hole at the duodenum close to the pylorus. The balloon was placed in the antrum to record the gastric motility. Subsequently, the hole was securely tied to avoid bloodletting and balloon shifting. Another end of the catheter was connected to a pressure transducer (TSD 104A; BIOPAC Systems, Inc., USA) via a three-way tube. The three-way tube was also connected to a syringe. The internal pressure of the balloon was maintained at 80–100 mmHg by injecting 0.3–0.4 ml of warm saline with the syringe. The pressure varied in accordance with the gastric motility changes. Therefore, it was an indicator of gastric motility. The pressure signal was recorded by the data acquisition system (MP150; BIOPAC Systems, Inc., USA) with the EGG transducer module. The acquisition parameter settings were LP: 1.0 Hz, HP: 0.005 Hz, and sampling frequency: 1000 Hz.

### 2.3. Vagal Afferent Recording

The vagal afferent recording was performed according to the protocol described by JN Sengupta [24] and under our laboratory conditions and using our equipment. Following the midline incision in the neck, after tracheal intubation, the skin was retracted using silk sutures tied to a stereotaxic frame to create a pool filled with warm mineral oil. The sternocleidomastoid, sternohyoid, and omohyoid were fixed to the skin with 502 glue to expose the right cervical vagus nerve. The vagus nerve was separated from the carotid artery and sympathetic nerve and was placed on a black microbase plate. After the perineural sheath removal, a small nerve bundle was teased off (3 mm in length) and split further to obtain single-unit recordings. The nerve action potentials were recorded by draping of the nerve fiber over the unipolar silver electrode (0.2 mm in diameter), while the ground electrode was pricked into the subcutaneous tissue to obtain single-end recordings. Action potentials were amplified using a low noise microelectrode amplifier (MCE 100C; BIOPAC Systems, Inc., USA) and recorded online using the MP system (MP150; BIOPAC Systems, Inc., USA). The parameter settings of amplifiers were as follows: GAIN: 1000, HP: 100 Hz, LP: 30 kHz, and sampling frequency: 25 kHz. Gastric motility and vagal afferent action potential were recorded continuously and synchronously in real time and were stored on the computer hard disk.

### 2.4. Defining Gastric Vagal Afferent

Another balloon (2 cm in diameter) attached to a catheter (2.3 mm OD and 1.3 mm ID) was inserted into the stomach through the mouth, esophagus, and cardia. The gastric vagal afferent fibers were initially identified by the balloon distention by injecting 10 ml air with a syringe. The positive response of the nerve fibers to distention is regarded as the activity of the gastric vagal afferent fibers [24].

### 2.5. The Model of Gastric Hypomotility

Gastric hypomotility was induced by injecting 0.01% atropine sulphate (0.25 ml/100 g) into the caudal vein. The injection speed was slow, as recommended, and finished in 1 min. The decrease in amplitude or frequency of gastric motility compared to the normal value or more than 25% of the normal state is considered a successful model of gastric hypomotility [25].

### 2.6. Acupuncture

An acupuncture needle (0.25 mm × 25 mm, Tianjin Hua Hong Medical Co., Ltd., Tianjin, China) was inserted vertically into the left ST36, located 3–4 mm below and 1–2 mm lateral from the midline of the knee [26]. After inducing de-qi sensation, four types of frequencies (1 Hz, 2 Hz, 3 Hz, and 4 Hz) of lifting-thrusting MA were performed on ST36 for 70 seconds in four MA groups, respectively. The procedure was performed by the same licensed acupuncture expert who used a metronome to keep the rhythm stable. Before application of MA on the rat, the acupuncturist practiced four types of MA manipulation using the ATP-II acupuncture manipulation parameter tester (which was manufactured by Shanghai University of Traditional Chinese Medicine, Shang Xin Medical Technology Company) to ensure that the amplitude, depth, and frequency of lifting-thrusting manipulation were stable and repeatable. Practice process and evaluation of MA were described previously by L-L Gao [23].

### 2.7. Experimental Procedure

**Figure 1:**
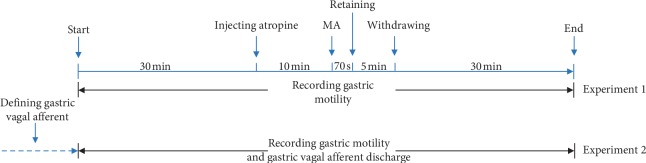
Schematic diagram of the experiments.

In the present study, two experiments were conducted subsequently. In the first experiment, the rats were divided into six groups, i.e., control, atropine, 1 Hz MA, 2 Hz MA, 3 Hz MA, and 4 Hz MA group. Normal gastric motility of the six groups was recorded for more than 30 min in each group in order to obtain a stable gastric motility waveform. Subsequently, the rats in the atropine group and four MA groups were injected with atropine into the caudal vein to induce gastric hypomotility, while the rats in the control group were injected with 0.9% normal saline. Ten minutes after the injection, the rats in the four MA groups were treated with lifting-thrusting MA manipulations with corresponding frequencies. In the control and atropine groups, no MA manipulations were applied. The period of stimulation of MA treatment was 70 seconds. After completed manipulation, the needle was retained for 5 min on the acupoint. The gastric motility was recorded during the entire procedure, 30 min after the needle removal. The regulating effects of MA treatment on gastric motility were compared among the six groups. In the second experiment, the aim was to investigate the mechanism of MA with different frequencies on the GVN discharge. After the completion of the first experiment, the frequency which was found to be the most effective and the one found to be the least effective were applied in the subsequent experiment. The rats were further divided into four groups (control, atropine, MA frequency with the greatest effect, and MA frequency with the least effect). The model of gastric hypomotility and MA manipulation were identical as mentioned above. The gastric motility and gastric vagal afferent discharge were recorded synchronously during the entire procedures. The schematic diagram of the study is shown in Figure 1. The total recording time was divided into six time intervals, including baseline, atropine injection, needling, and 10, 20, and 30 min after withdrawing the needle. For each time interval, relatively stable gastric motility waves for 5 min were extracted to calculate the average frequency and amplitude values. The gastric vagal afferent discharge values corresponding to the gastric motility waveform were extracted to calculate the average discharge value.

### 2.8. Data Analysis

The change rates of the frequency and amplitude of gastric motility and discharge frequency of the gastric vagal afferent fibers were analyzed in each group and were calculated by the following formula: (the average value of each time point − the average value of baseline)/the average value of baseline × 100%.

Data were expressed as means ± standard error of the mean (SEM). Within each group, the six time points were compared by repeated measures analysis of variance (ANOVA). Between-group comparisons were made using one-way ANOVA followed by post hoc, least significant difference test. A *p* value less than 0.05 was regarded as statistically significant. Statistical testing was performed with SPSS19.0 (SPSS Inc. Chicago, IL, USA).

## 3. Results

### 3.1. The Effects of Gastric Motility Regulation by MA with Frequencies of 1 Hz, 2 Hz, 3 Hz, and 4 Hz

**Figure 2:**
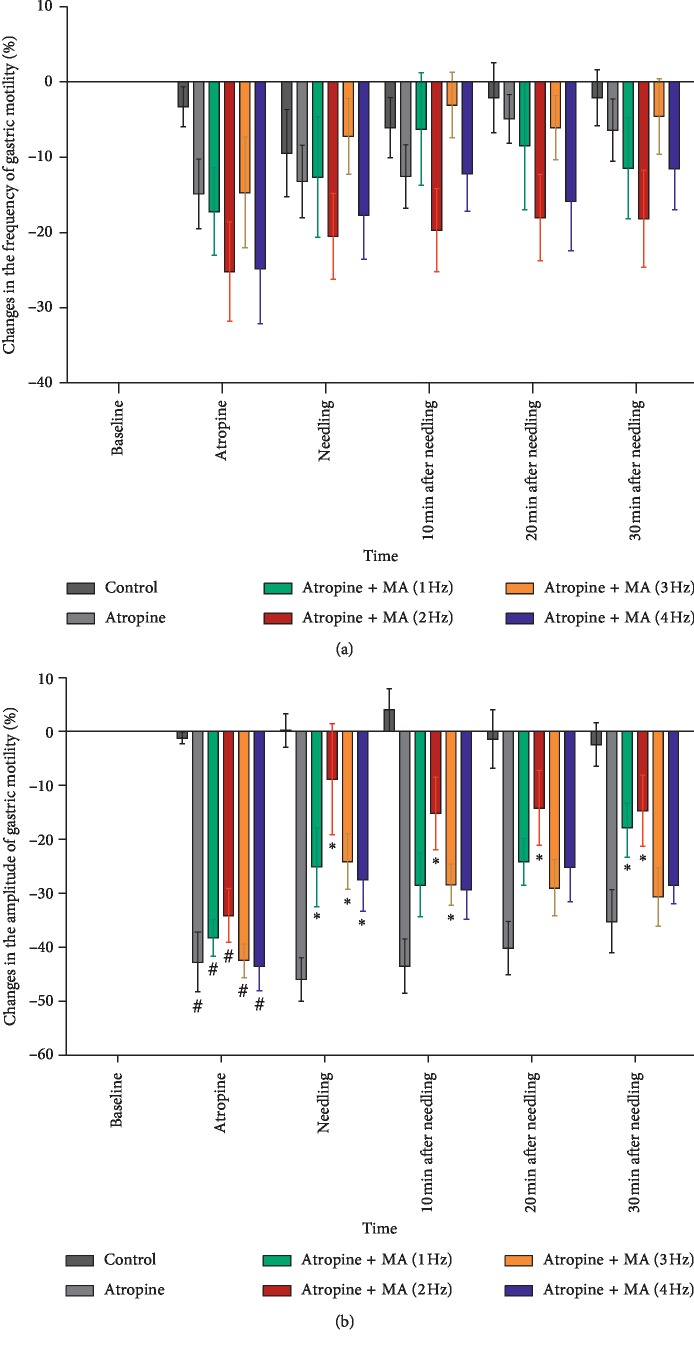
Gastric motility responses to atropine and MA with different frequencies (1 Hz, 2 Hz, 3 Hz, and 4 Hz) at ST36. (a, b) Changes in the frequency and amplitude of gastric motility induced by atropine and different MA simulations at ST36, respectively (normal, model, 1 Hz, 2 Hz, 3 Hz, and 4 Hz; *n* = 5, 6, 5, 6, 6, and 6, respectively; ^#^*p* < 0.05 versus the normal group at the indicated time point; ^*∗*^*p* < 0.05 versus the atropine group at the indicated time point).

As shown in Figure 2(a), there was no significant change in the gastric motility frequency among groups during the time of observation (*p* > 0.05) and no significant differences were found between the groups at any time point (*p* > 0.05). Although the gastric motility frequency decreased after atropine injection, it had no statistical difference (*p* > 0.05). MA with four types of frequencies on ST36 had no significant effect on the gastric motility frequency after atropine injection.

The changes in the gastric motility amplitude are presented in Figure 2(b). In the control group, no significant change of gastric motility amplitude was found, while the amplitude significantly decreased after atropine injection in the atropine and four atropine + MA groups (control group: −1.2 ± 1.1%; atropine group and four MA groups (1 Hz MA, 2 Hz MA, 3 Hz MA, and 4 Hz MA): −43.0 ± 5.6%, −38.4 ± 3.4%, −34.1 ± 5.0%, −42.7 ± 3.1%, and −43.7 ± 4.3%, respectively, all *p* < 0.01). There were no significant differences in amplitude after atropine injection between the atropine and atropine + MA groups.

In the four MA groups, the gastric motility amplitude increased after four types of MA manipulation on ST36 in the rats following atropine injection within each group. In comparison to the atropine group at the needling time point (−43.0 ± 5.6%), the changes in the amplitude in MA groups with the frequencies of 1 Hz, 2 Hz, 3 Hz, and 4 Hz increased for −25.2 ± 7.3% (*p*=0.034), −8.8 ± 8.2% (*p* ≤ 0.001), −24.2 ± 5.2% (*p*=0.02), and −27.7 ± 5.7% (*p*=0.048), respectively; and the MA with 2 Hz was found to be the most effective. After the needle removal, the amplitude in the four MA groups remains higher than in the atropine group. MA of 2 Hz intensity showed a significant difference compared to the atropine group at three indicated time points after needling (10 min after needling: −43.6 ± 3.0% vs −15.2 ± 6.7%, *p*=0.001; 20 min after needling: −40.3 ± 5.0% vs −14.2 ± 6.9%, *p*=0.003; and 30 min after needling: −35.3 ± 5.9% vs −14.7 ± 6.6%, *p*=0.007). MA of 1 Hz showed a significant difference at 30 min after needling compared to the atropine group (−17.8 ± 4.5%, *p*=0.027), and MA of 3 Hz showed a significant difference at 10 min after needling compared to the atropine group (−28.5 ± 3.9% vs −15.2 ± 6.7%, *p*=0.048), while MA of 4 Hz had no significant difference compared to the atropine group at any time point after needle withdrawal. The result indicated that the MA with four different frequencies of 1 Hz, 2 Hz, 3 Hz, and 4 Hz was able to recover the gastric motility amplitude in gastric hypomotility rats induced by atropine. The MA with a frequency of 2 Hz showed the highest amplitude regulation effect, while the frequency of 4 Hz was found to be the least effective among the four types of MA. Therefore, the MA with the frequency of 2 Hz and 4 Hz were used in the second experiment to clarify the mechanism of the gastric vagal afferent fiber activity.

### 3.2. The Effects on Gastric Motility Regulation and Discharge Frequency of Vagal Afferent Fibers by MA with a Frequency of 2 Hz and 4 Hz

**Figure 3:**
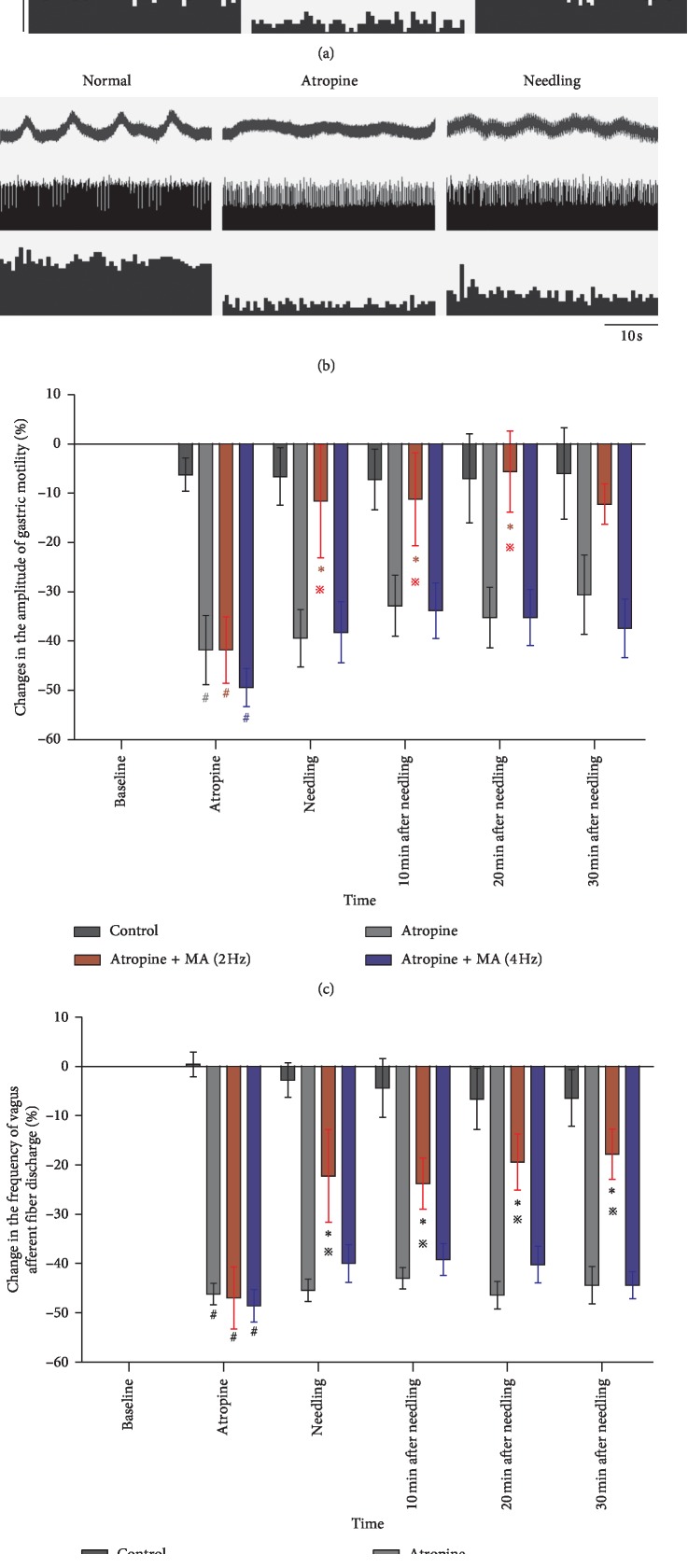
Responses of gastric motility and gastric vagal afferent fiber discharge due to atropine and MA treatment with different frequencies (2 Hz and 4 Hz) at ST36. (a, b) Examples of the alterations of gastric motility and gastric vagal afferent fiber discharge induced by atropine and MA stimulations with different frequencies (2 and 4 Hz) at ST36. In each panel, the top trace is the wave of gastric motility, the middle trace is the action potentials of the gastric vagal afferent fibers, and the bottom trace represents the PSTH (1 s bin width). (c, d) Changes in the gastric motility and vagal afferent fibers after the atropine injection and MA stimulation at ST36 (normal, model, 2 Hz, and 4 Hz, and *n* = 5, respectively; ^#^*p* < 0.05 versus the normal group at the indicated time point; ^*∗*^*p* < 0.05 versus the atropine group at the indicated time point; ※*p* < 0.05 versus the 4 Hz group after modeling).

The gastric motility and vagal afferent fibers were recorded synchronously. Figures 3(a) and 3(b) show responses of gastric motility and vagal afferent fibers after the atropine injection and MA stimulation with a frequency of 2 Hz and 4 Hz, respectively. The changes in the gastric motility amplitude due to MA presented in Figure 3(c) were similar to the changes in the discharge frequency of gastric vagal afferent fibers caused also by MA (Figure 3(d)). The amplitude of gastric motility and discharge frequency of vagal afferent fibers decreased significantly after the atropine injection (all *p* < 0.01). MA with a frequency of 2 Hz was able to increase the amplitude during the needling and after needle removal compared to the atropine group (needing time: −11.6% ± 11.47% vs −39.4 ± 5.8%, *p*=0.022; 10 min after needling: −11.2% ± 9.4% vs −32.8% ± 6.2%, *p*=0.045; and 20 min after needling: −5.6% ± 8.2% vs −35.2% ± 6.1%, *p*=0.012). However, MA with a frequency of 4 Hz had no significant effects on gastric motility. Correspondingly, MA with a frequency of 2 Hz increased the discharge frequency of vagal afferent fibers compared to the atropine group (needling: −22.2% ± 9.4% vs −45.4% ± 2.2%, *p*=0.09; 10 min after needling: −23.8% ± 5.2% vs −43% ± 2.2%, *p*=0.007; 20 min after needling: −19.4% ± 5.7% vs 46.4% ± 2.8%, *p*=0.001; and 30 min after needling: −17.8% ± 5.1% vs −44.4% ± 3.8, *p*=0.001), while MA with a frequency of 4 Hz did not show a significant increasing effect on vagal afferent discharge.

## 4. Discussion

In this study, the regulation effects of MA treatment on the gastric motility in rats with gastric hypomotility were investigated, applying four different frequencies (1 Hz, 2 Hz, 3 Hz, and 4 Hz) on ST36. The results showed that the amplitude of gastric motility can be increased by applying lifting-thrusting MA at ST36 after gastric hypomotility induced by injecting atropine in rats, which is consistent with the previous studies [27, 28]. Moreover, MA with four types of frequencies of 1 Hz, 2 Hz, 3 Hz, and 4 Hz showed different recovery effects on gastric hypomotility rats regarding the gastric motility amplitude. Specifically, MA with the frequency of 2 Hz was found to be the most effective, while 4 Hz frequency showed the least effectiveness; the effects of 1 Hz and 3 Hz frequencies were between the effects induced with 2 Hz and 4 Hz. These results indicate the presence of an “intensity (frequency)-response” relationship of MA. Therefore, it is necessary to determine the optimal parameters of MA to improve the treatment effect in acupuncture clinical practice. In our previous study, a similar “intensity-response” relationship was found using twirling manipulations [23]. Lifting-thrusting and twirling are two different acupuncture manipulations, which both showed that the gentle stimulation induced better effects on the gastric motility recovery than strong manipulation. These results indicate the greater importance of acupuncture intensity (frequency) compared to the variation of the acupuncture manipulations employed.

Therefore, further research is necessary in order to determine whether the gentle MA is more effective than the strong MA in other diseases or conditions. Nevertheless, in our previous study, it was found that the strong MA exhibited greater effects on acute visceral nociception than the gentle MA [29]. Tian et al. [30] study showed that high-frequency electroacupuncture induced inhibiting effects on the activation of abnormal astrocytes to a greater extent compared with low-frequency electroacupuncture through down-regulation of COX-2, *β*-catenin, and NK-1R expression, and thus alleviating the inflammation and poststroke pain. These findings suggest that MA with different frequencies at ST36 may induce various modulatory effects depending on different diseases. Besides, optimal stimulation parameters of MA may vary according to certain diseases or conditions.

The gastric function is mainly regulated by the vagus nerve; therefore, in our second experiment, the response of gastric vagal afferent fibers to MA treatment with different frequencies on ST36 was examined in order to investigate the mechanism of MA regulation of gastric motility. Simultaneously, the gastric motility was observed. The results of the first experiment showed that MA of 2 Hz was the most effective frequency for the gastric motility improvement, while 4 Hz showed the least effectiveness. Therefore, the MA of 2 Hz and 4 Hz were applied in the subsequent experiment, where the result showed that the changes in the discharge frequency of gastric vagal afferent fibers were similar to the changes in the gastric motility amplitude. Specifically, when the gastric motility amplitude decreased due to atropine injection, the discharge frequency of gastric vagal afferent fibers decreased as well. Furthermore, when the gastric motility amplitude increased after MA at ST36, the discharge frequency of gastric vagal afferent fibers also increased. Moreover, MA of 2 Hz induced the most effective results on the gastric motility, likewise the 2 Hz frequency MA on the discharge amplitude of gastric vagal afferent fibers. In addition, no significant effect of 4 Hz MA was recorded on the gastric motility amplitude or the 4 Hz MA on the discharge frequency of the gastric vagal afferent fibers. The result indicated that the gastric motility regulated by MA was associated with vagal afferent activation.

In this study, the discharge of vagal efferent fibers was not recorded; however, it is known that vagal efferent nerves regulate gastric function. Furthermore, it was reported that the gastric motility was excited by acupuncture-like stimulation applied to the hind paw of rats, followed by an increase in the activity of the gastric vagal efferent nerves [31]. Thus, it is of great importance to investigate whether the acupuncture on ST36 may increase the vagal efferent discharge, which may further promote the recovery of gastric hypomotility. This mechanism will be investigated in the future study. Besides, the dorsal vagal complex, which is the central part of the vagus nerve, may also have roles in the gastric motility regulation induced with acupuncture. A large number of studies have confirmed that acupuncture is able to activate the nucleus tractus solitari (NTS) and the dorsal motor nucleus of the vagus (DMV) [32–34]. We presumed that one of the super-spinal mechanisms of gastric regulation is that the gastric information reaches to the NTS through the vagus afferent nerve, integrating with the information from acupuncture and then transmits to the DVM, which regulates gastric function through the vagal efferent nerves. The variation of MA effects may partly be a result of different ways of the vagus nerve activation.

In this study, the gastric hypomotility was induced with atropine, a muscarinic acetylcholine (Ach) receptor antagonist, which induces gastric hypomotility by blocking of Ach binding to M receptor. In the atropine group (model group), the decreased gastric motility was followed by a spontaneous recovery to a certain degree, which might be associated with the metabolism of atropine and eventual binding of Ach to M receptor; however, MA was able to accelerate the recovery of gastric motility. Furthermore, Xinyan Gao's [35] study revealed that ACh M2/3 receptors are required for the enhancement of the gastric motility induced with acupuncture at ST36. Thus, the assumed mechanism of MA effects on the gastric motility is due to activation of the vagus system and release of additional Ach or M receptor.

## 5. Conclusion

The manual acupuncture with lifting-thrusting manipulation at ST36 successfully recovered the gastric motility amplitude in rats with gastric hypomotility induced by atropine. Moreover, the variation of MA intensity (frequency) produced different effects. The mechanism of the gastric motility regulation by MA was associated with vagus afferent activation. Further research should be conducted in order to determine the optimal stimulation parameters of MA, which vary according to certain diseases or conditions.

## Data Availability

The data used to support the findings of this study are available from the corresponding author upon request.
